# Synergistic effect of oridonin and a PI3K/mTOR inhibitor on the non-germinal center B cell-like subtype of diffuse large B cell lymphoma

**DOI:** 10.1186/s13045-016-0303-0

**Published:** 2016-08-23

**Authors:** Kai Qing, Zhen Jin, Wanbin Fu, Wenfang Wang, Zhao Liu, Xiaoyang Li, Zizhen Xu, Junmin Li

**Affiliations:** 1 Shanghai Institute of Hematology, State Key Laboratory for Medical Genomics, Ruijin Hospital affiliated to School of Medicine, Shanghai Jiao Tong University, Shanghai, China; 2 Department of Hematology, Shanghai Institute of Hematology, Ruijin Hospital affiliated to School of Medicine, Shanghai Jiao Tong University, 197 Rui Jin Er Road, Shanghai, China; 3 Department of Laboratory Medicine, Ruijin Hospital affiliated to School of Medicine, Shanghai Jiao Tong University, 197 Rui Jin Er Road, Shanghai, China

**Keywords:** Diffuse large B cell lymphoma, Oridonin, NVP-BEZ235, Apoptosis, PI3K/mTOR, NF-kB

## Abstract

We demonstrate the synergistic antitumor effect of oridonin and the PI3K/mTOR inhibitor NVP-BEZ235 on the non-germinal center B cell-like subtype of diffuse large B cell lymphoma (non-GCB DLBCL) both in vitro and in vivo. The underlying mechanism may be multifunctional, involving apoptosis, AKT/mTOR and NF-kB inactivation, and ROS-mediated DNA damage response. Our findings pave the way for a new potential treatment option for non-GCB DLBCL with the combination of oridonin and NVP-BEZ235.

**Electronic supplementary material:**

The online version of this article (doi:10.1186/s13045-016-0303-0) contains supplementary material, which is available to authorized users.

## Findings

Diffuse large B cell lymphoma (DLBCL) is the most common aggressive form of non-Hodgkin’s lymphoma (NHL) in adults, and it can be distinguished into two major groups, the germinal center B cell (GCB) subtype and the non-germinal center B cell-like (non-GCB) subtype [1, 2]. The non-germinal center B cell-like subtype of diffuse large B cell lymphoma (non-GCB DLBCL) presents aggressive clinical courses and poor prognosis [3, 4]. Targeting key pathways may raise the possibility of improving clinical outcomes.

Our previous studies have indicated that oridonin and NVP-BEZ235 have some antitumor effects in DLBCL cells [5, 6]. The aim of this study was to determine whether oridonin combined with NVP-BEZ235 could achieve a more significant antitumor effect on the non-GCB DLBCL, and to further investigate the underlying mechanism. The materials and methods used in this study are detailed in Additional file 1.

**Fig. 1:**
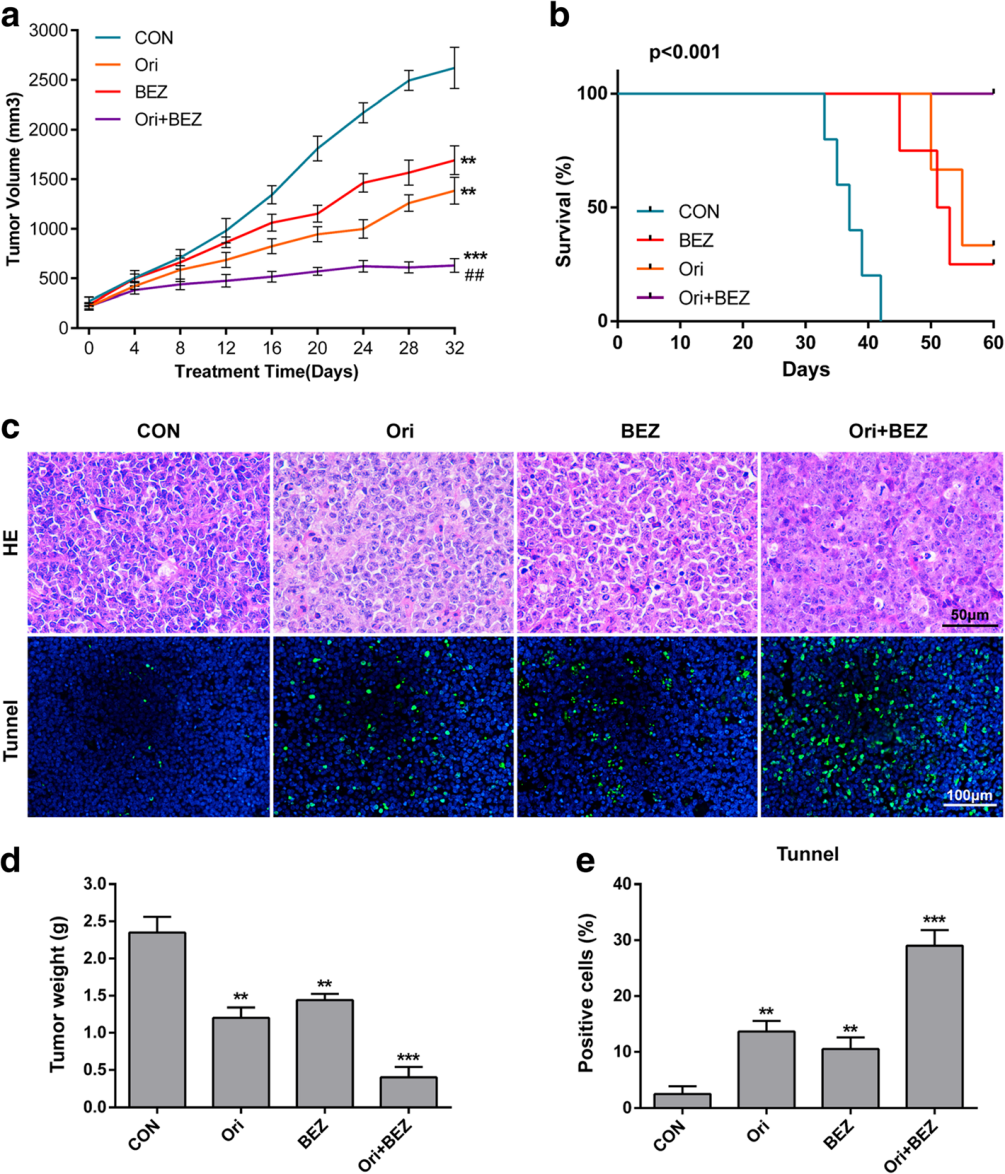
Oridonin combined with NVP-BEZ235 dramatically inhibited tumor growth and prolonged the survival in a non-GCB DLBCL xenograft mouse model (SU-DHL-2). **a, d** Mice in each cohort were treated with oridonin (5 mg/kg) and NVP-BEZ235 (20 mg/kg) alone or in combination every other day. Tumor volumes were measured once every 4 days. After 32 days, the mice were sacrificed, and the tumors were removed and weighed. **b** Overall survival was prolonged by oridonin and NVP-BEZ235 combination therapy compared with the control group and single-agent group (*p* < 0.005). **c** HE staining and TUNEL assay was performed to examine the apoptosis in tumor tissues. **e** *Bar graph* illustrate the proportion of positive cells showed in TUNEL assay. *p < 0.05, **p <0.01, ***p < 0.001 compared with the control group; ^#^ *p* < 0. 05, ^##^ *p* < 0.01 compared with single-agent group

Our results demonstrate that oridonin and NVP-BEZ235 exhibit a synergistic effect on non-GCB DLBCL cell lines (OCI-Ly3 and SU-DHL-2), and the co-treatment was more effective on cell proliferation inhibition compared with single-agent therapy (Additional file 2). And then cytotoxic effect of oridonin and NVP-BEZ235 alone or in combination were evaluated in nude mice bearing SU-DHL-2 tumors. Compared with the control group or single-agent group, the co-treatment group exhibited more significant DLBCL cell growth inhibition in terms of tumor size (Fig. 1a) and weight (Fig. 1d) and prolonged the mice survival (Fig. 1b). H&E staining and TUNEL assay showed that co-treatment with oridonin and NVP-BEZ235 obviously increased apoptosis (Fig. 1c, e).

**Fig. 2:**
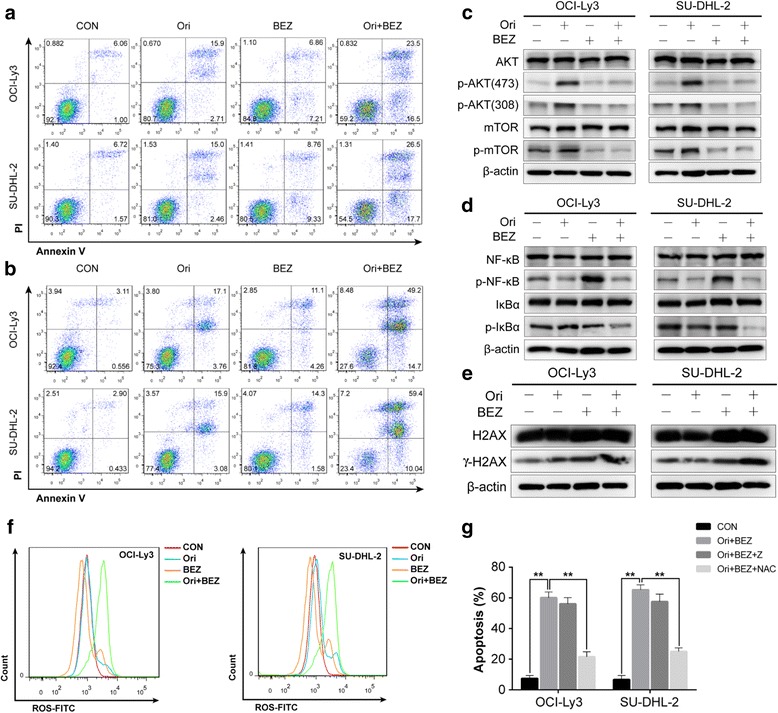
The mechanism of synergistic antitumor effect of oridonin and NVP-BEZ235 on non-GCB DLBCL. **a, b** Cell lines were treated with oridonin (2 μM) and NVP-BEZ235 (25 nM) alone or in combination for 24 and 48 h, analyzing apoptosis by Annexin-V/PI staining. **c** Cell lines were treated with oridonin (2 μM) and NVP-BEZ235 (25 nM) alone or in combination for 48 h. Western blot analysis was performed to identify the expression of total AKT, p-AKT (Ser473), p-AKT (Thr308), mTOR, and p-mTOR. **d** Western blot for NF-kB, p-NF-kB, IkBα, and p-IkBα. **e** The expression of γH2AX and H2AX was analyzed by western blotting. **f** Cell lines were simultaneously treated with oridonin (2 μM) and NVP-BEZ235 (25 nM) for 48 h, and FACS quantitative analysis of DCF-DA was used to detect the expression of ROS. **g** Pretreatment of co-treatment group cells with NAC (5 mM) and Z-DEVD-FMK (10 μM), respectively, for 48 h, analyzing apoptosis by Annexin-V/PI staining with *t* test statistic assay. (Mean ± SD, *n* = 3, *p < 0.05, **p < 0.01 compared with Ori + BEZ group. *Z*: Z-DEVD-FMK)

To explore the mechanism underlying the synergistic antitumor effect, both cell lines were exposed to 2 μM oridonin and 25 nM NVP-BEZ235 alone or in combination for 24 and 48 h. The results showed that the co-treatment induced higher apoptosis in non-GCB DLBCL cell lines (Fig. 2a, b). Meanwhile, the apoptosis induced by the drug combination was further confirmed by assessment of caspase family and Bcl-2 family protein expression (Additional file 3). However, co-treatment does not further enhance cell-cycle arrest in G0/G1 phase (Additional file 4).

To further investigate the mechanism of synergistic drug effects, the expression of AKT/mTOR and NF-kB pathway was assessed by western blotting. The data suggest that the simultaneous inhibition of the PI3K/AKT/mTOR and NF-kB pathways abrogated the key survival signals for non-GCB DLBCL, which might indicate the mechanism for the synergistic pro-apoptotic effect of oridonin and NVP-BEZ235 in combination (Fig. 2c, d). We then found that oridonin and NVP-BEZ235 in combination markedly induced the expression of γH2AX, a marker of DNA damage. Flow cytometry also demonstrated a significant increase in reactive oxygen species (ROS) (Fig. 2e, f). Addition of *N*-acetyl-l-cysteine (NAC) largely reversed co-treatment-induced apoptosis, while treating the cells with Z-DEVD-FMK (caspase 3 inhibitor) had little effect (Fig. 2g, Additional file 5).

Taken together, our findings demonstrated the synergistic antitumor effect of oridonin combined with NVP-BEZ235 in non-GCB DLBCL cell lines. The potential molecular mechanism might be multifunctional, involving apoptosis, AKT/mTOR and NF-kB inactivation, and ROS-mediated DNA damage response. Moreover, co-treatment was also effective in a non-GCB DLBCL xenograft model. Therefore, our study provides a theoretical basis and preclinical evidence for this novel strategy and suggests that the combination of oridonin and NVP-BEZ235 might have promising therapeutic application in non-GCB DLBCL patients.

## Additional files

Additional file 1: Materials and methods. (PDF 106 kb) Additional file 2: Combination effects of oridonin and NVP-BEZ235 on proliferation inhibition. (A) Cell viability of OCI-Ly3, SU-DHL-2 was measured by CCK-8 assay after treated with oridonin (2 μM) and NVP-BEZ235 (25 nM) alone or in combination for 48 h. Drugs were conducted simultaneously at the indicated concentrations. (B) Isobologram analysis of agent combination at ED50 and CI values were calculated as well. (C) CI values of all cell lines at ED25, ED50, ED75, and ED90. The lower CI values indicated the stronger synergism when CI < 1, and higher CI values means the stronger antagonism when CI > 1. Additive effect was designated with CI = 1. (PDF 229 kb) Additional file 3: Oridonin combined with NVP-BEZ235 significantly increased the apoptosis on non-GCB DLBCL cell lines. (A, B) Cell lines were simultaneously treated with oridonin (2 μM) and NVP-BEZ235 (25 nM) for 24 and 48 h, analyzing apoptosis by Annexin-V/PI staining with *t* test statistic assay. (Mean ± SD, *n* = 3, **p* < 0.05, ***p* < 0.01, ****p* < 0.001 compared with control group; ^#^ *p* < 0. 05, ^##^ *p* < 0.01 compared with single agent group. (C) Cell lines were subjected to indicate treatments and protein lysates were performed with immunoblotting, incubating with PARP, caspase3, caspase9, cleaved-PARP, cleaved-caspase3, cleaved-caspase9, Bax and Bcl-2 antibodies. (PDF 191 kb) Additional file 4: The effect of oridonin and NVP-BEZ235 on cell cycle. (A) Cells were treated with drugs for 48 h with the dosages indicated in Fig 2. Cell cycle assay was assessed with cytometry and Modfit LT. The percentage of G0 and G1phase cells is respectively shown in the left bottom and left top corner of each panel. (B) The number of G0/G1 cells was determined using quantitative FACS analysis. Each column represents the mean ± SD (*n* = 3). Statistical analysis was performed using the Student’s *t* test. *NS* not significant; **p* < 0.05, ***p* < 0.01 compared with the control group. (PDF 295 kb) Additional file 5: Co-treatment triggered ROS generation which mediated non-GCB DLBCL cell apoptosis. (A) Cell lines were simultaneously treated with oridonin (2 μM) and NVP-BEZ235 (25 nM) for 48 h, FACS quantitative analysis of DCF-DA was used to detect ROS with *t* test statistic assay. (Mean ± SD, *n* = 3, **p* < 0.05, ***p* < 0.01 compared with control group. (B) NAC pretreatment attenuated co-treatment-induced protein expression levels of γH2AX, cleaved-caspase 3 and cleaved-caspase 9. (C) Pretreatment of co-treatment group cells with NAC (5 mM) and Z-DEVD-FMK (10 μM), respectively, for 48 h, analyzing apoptosis by Annexin-V/PI staining. (PDF 404 kb)

## Abbreviations

DLBCL: Diffuse large B cell lymphoma
ROS: Reactive oxygen species
non-GCB: Non-germinal center B cell-like
GCB: Germinal center B cell
OS: Overall survival
PFS: Progression free survival
R-CHOP: Rituximab, cyclophosphamide, doxorubicin, vincristine and prednisolone
NF-kB: Nuclear factor kappa B
Ori: Oridonin
BEZ: NVP-BEZ235
NAC: *N*-acetyl-l-cysteine
Tunel: Terminal deoxytransferase-catalyzed DNA nick-end labeling
H&E: Hematoxylin and eosin
