# Supplementary material for: Synergistic effect of oridonin and a PI3K/mTOR inhibitor on the non-germinal center B cell-like subtype of diffuse large B cell lymphoma
Source: J Hematol Oncol. 2016 Aug 23;9(1):72. doi: 10.1186/s13045-016-0303-0 (PMC4995739; doi:10.1186/s13045-016-0303-0)
Supplement: Additional file 1: — Materials and methods. (PDF 106 kb) [file 13045_2016_303_MOESM1_ESM.pdf]

Additional file 1: Materials and methods.

Cells and cell culture

The non-GCB DLBCL-derived cell lines OCI-Ly3 and SU-DHL-2 were obtained from Dr. T Zhao (Nanfang Hospital affiliated to Southern Medical University, China). Cell lines were cultured in IMDM (Invitrogen) with 10% FBS (Invitrogen) and incubated at 37°C with 5% CO_2.

Cell proliferation assays

Cell proliferation analysis was performed using the Cell Counting Kit-8 (Dojindo, Japan) assay. Ori was purchased from PureOne Biotechnology (Shanghai, China), NVP-BEZ235 was purchased from Selleck (Huston, TX, USA), and both were dissolved in DMSO. Ori treatment was administered at 0.25 μM, 0.5 μM, 1 μM, 2 μM and 4 μM, and the working concentration of BEZ235 was 6.25 nM, 12.5 nM, 25 nM, 50 nM and 100 nM. The cells were seeded in a 96-well plate at a concentration of 1×10^5/mL. After 48 h, 10 μL Cell Counting Kit-8 reagent was added to each well and incubated for 2-3 h. The absorbance at 450 nm was measured using a microplate reader. Growth inhibition was calculated with the formula (OD absorbance of treatment group–OD absorbance of blank)/(OD absorbance of control group–OD absorbance of blank)×100%. The synergetic effect of two drugs was measured using the combination index (CI) using CalcuSyn software (Version 2.1). CI<1 indicates the synergetic effect, CI=1 indicates an additive effect, and CI>1 suggests antagonism.

Western blotting

Antibodies against Bax, Bcl-2, Caspase-3, cleaved Caspase-3, Caspase-9, cleaved

Caspase-9, PARP, cleaved PARP, H2AX, γH2AX, AKT, p-AKT(473), p-AKT(308), mTOR, p-mTOR, NF-κB, p-NF-κB, IκBα, and p-IκBα were obtained from Cell Signaling Technology. All of the cell lines were treated with Ori and NVP-BEZ235 singly or in combination for 48 h. Samples from each group were then collected, resolved using standard SDS-PAGE gel electrophoresis, and transferred to PVDF membranes. After blocking with 5% non-fat milk in 0.1 % TBST, the membrane was probed with primary antibodies overnight at 4°C. After washing with TBST, the membrane was incubated with HRP-conjugated secondary antibodies (Santa Cruz, CA) for 1 h. The bands were detected and quantified using Image Lab software (Bio-Rad Laboratories, California, USA).

Apoptosis assays

Apoptosis was assessed using flow cytometry according to the FITC Annexin V Apoptosis Detection Kit I protocol (BD Bioscience). Cells were collected and washed in cold phosphate-buffered saline (PBS), then resuspended in Annexin-binding buffer and stained with propidium iodide (PI) and FITC Annexin V. After incubation in the dark at room temperature for 15 min, the cell suspensions were diluted in Annexin-binding buffer and analyzed using BD LSRFORTESSA flow cytometry immediately. The data were acquired using FlowJo software (Tree Star).

Cell cycle assay

Each cell line was seeded into a 12-well plate and exposed to drugs for 48 h. Then, the cells were collected, centrifuged, washed in ice-cold PBS and resuspended with 70% ethanol. After fixation at -20°C for 12 h, the cells were resuspended in PBS containing

20 μg/mL Ki67 and 20 μg/mL Hoechest33342 30 min prior to analysis. Cell cycle data were assessed using BD LSRFORTESSA flow cytometry and analyzed with FlowJo software (Tree Star). G0%, G1% and S/G2/M% were calculated to determine the distribution of cells in these phases.

Flow cytometry assays to detect reactive oxygen species (ROS)

Measurements of ROS production were performed using ROS Detection Reagents (Invitrogen) according to the manufacturer's instructions. The cells were washed in PBS and incubated with 300 nM 2',7'-dicholorodihydrofluorescein diacetate (DCF-DA) for 20 min at 37°C. The fluorescent intensity was measured using BD LSRFORTESSA and analyzed with FlowJo software (Tree Star).

Mouse model

Four- to six-week-old female nude mice purchased from Shanghai Laboratory Animal Center (Shanghai, China) were injected subcutaneously in the right flank with 1×10^7 SU-DHL-2 cells in 100 μL PBS. When the tumor volume reached approximately 150–300 mm^3, mice were randomly divided into 4 groups with 5 mice per group as follows: (I) control: intraperitoneally injected with saline; (II) Ori: intraperitoneally injected Ori at 5 mg/kg (diluted in 4 % DMSO, 96 % saline) every other day; (III) NVP-BEZ235: BEZ235 20 mg/kg by oral gavage dissolved in 10% NMP (1-methyl-2-pyrrolidone)/PEG 300 90% every other day; (IV) combination: treatment with Ori and NVP-BEZ235. Tumor volumes were measured every four days with a vernier caliper for 32 days and were calculated as $V=a \times b^2/2$ (a, the length of tumor; b, the width of tumor). At the end of the observation period, the mice were sacrificed, and the tumors

were harvested. Then, the samples were fixed in 4% formalin and processed for frozen sectioning. The animal protocol was approved by the Animal Care and Use Committee of Ruijin Hospital affiliated to the School of Medicine, Shanghai Jiao Tong University.

Hematoxylin and eosin staining

Tumor tissues were immersed in 4% paraformaldehyde for 4 h, then transferred to 70% ethanol. Individual lobes from tumor tissue biopsies were placed in processing cassettes, dehydrated through a serial alcohol gradient, and embedded in paraffin wax blocks. Before immunostaining, 5-μm-thick tumor tissue sections were dewaxed in xylene, rehydrated through decreasing concentrations of ethanol, and washed in PBS. Next, the samples were stained with hematoxylin and eosin (H&E). After staining, the sections were dehydrated through increasing concentrations of ethanol and xylene. Finally, one or two drops of mounting medium was added, and a coverslip was placed on top.

Terminal deoxytransferase-catalyzed DNA nick-end labeling (Tunel) assay

Frozen sections were stained with a terminal transferase recombinant kit (Roche, Indianapolis, IN, USA) using the manufacturer’s protocol. Cells with green fluorescence were classified as apoptotic. Image-Pro plus 6.0 (Media Cybernetics, USA) was used to identify the positive cells on each slide.

Statistical analysis

All of the data in this study were analyzed with SPSS 17.0 software (Chicago, USA) and presented as the mean ± standard deviation. Independent t-tests were used to identify significant differences between groups. IC_{50} values were analyzed using a probit assay in SPSS. $P<0.05$ was considered statistically significant. Overall survival

was measured with the Kaplan–Meier method. The drug interactions were stated according to the Chou–Talalay method and isobologram analysis.
