# Supplementary material for: Synergistic effect of oridonin and a PI3K/mTOR inhibitor on the non-germinal center B cell-like subtype of diffuse large B cell lymphoma
Source: J Hematol Oncol. 2016 Aug 23;9(1):72. doi: 10.1186/s13045-016-0303-0 (PMC4995739; doi:10.1186/s13045-016-0303-0)
Supplement: Additional file 2: — Combination effects of oridonin and NVP-BEZ235 on proliferation inhibition. (A) Cell viability of OCI-Ly3, SU-DHL-2 was measured by CCK-8 assay after treated with oridonin (2 μM) and NVP-BEZ235 (25 nM) alone or in combination for 48 h. Drugs were conducted simultaneously at the indicated concentrations. (B) Isobologram analysis of agent combination at ED50 and CI values were calculated as well. (C) CI values of all cell lines at ED25, ED50, ED75, and ED90. The lower CI values indicated the stronger synergism when CI < 1, and higher CI values means the stronger antagonism when CI > 1. Additive effect was designated with CI = 1. (PDF 229 kb) [file 13045_2016_303_MOESM2_ESM.pdf]

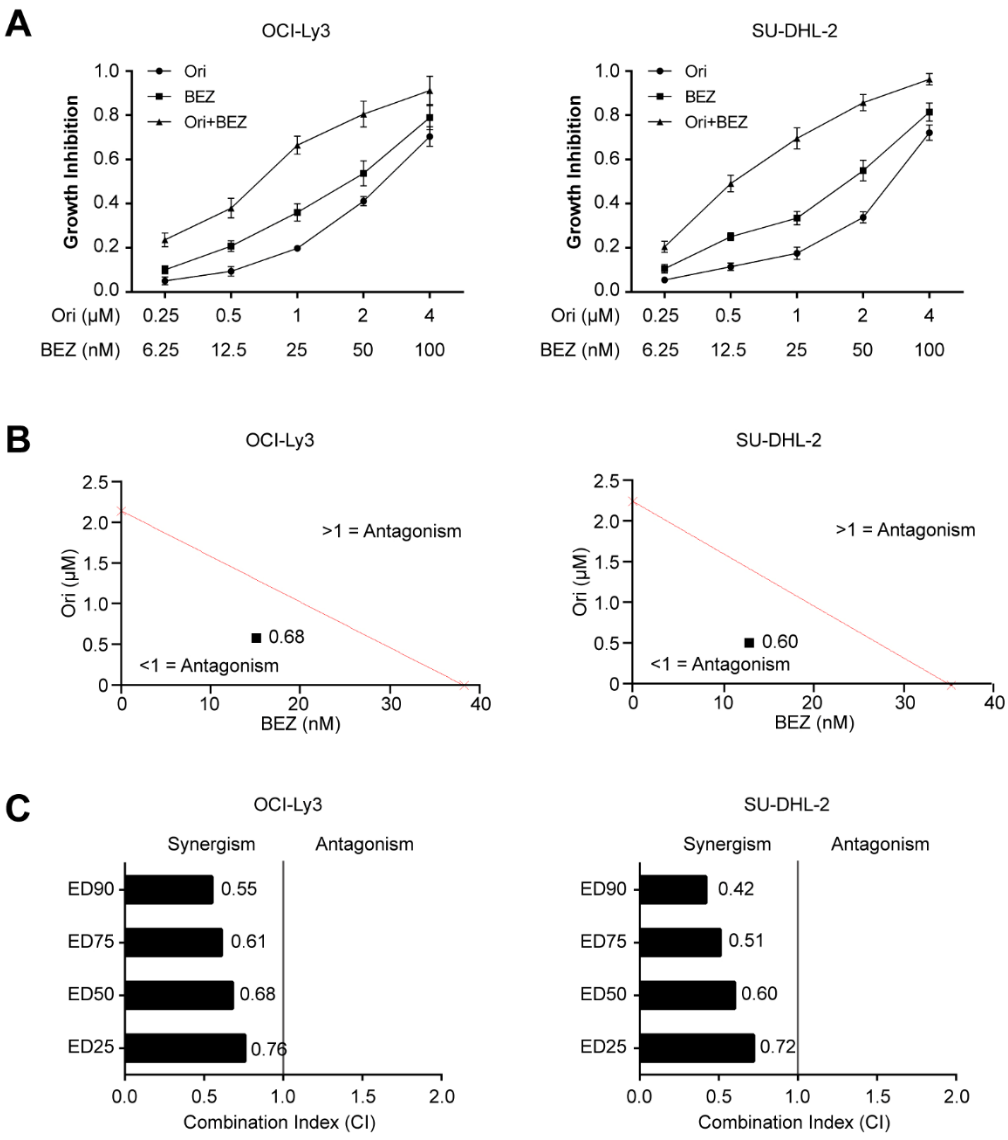

Additional file 2: Combination effects of oridonin and NVP-BEZ235 on proliferation inhibition.

(A) Cell viability of OCI-Ly3, SU-DHL-2 was measured by CCK-8 assay after treated with oridonin (2 μM) and NVP-BEZ235 (25 nM) alone or in combination for 48h. Drugs were conducted simultaneously at the indicated concentrations. **(B)** Isobologram analysis of agent combination at ED50 and CI values were calculated as well. **(C)** CI values of all cell lines at ED25, ED50, ED75 and ED90. The lower CI values indicated the stronger synergism when CI < 1, and higher CI values means the stronger

antagonism when CI >1. Additive effect was designated with CI = 1.
