# Supplementary material for: Synergistic effect of oridonin and a PI3K/mTOR inhibitor on the non-germinal center B cell-like subtype of diffuse large B cell lymphoma
Source: J Hematol Oncol. 2016 Aug 23;9(1):72. doi: 10.1186/s13045-016-0303-0 (PMC4995739; doi:10.1186/s13045-016-0303-0)
Supplement: Additional file 3: — Oridonin combined with NVP-BEZ235 significantly increased the apoptosis on non-GCB DLBCL cell lines. (A, B) Cell lines were simultaneously treated with oridonin (2 μM) and NVP-BEZ235 (25 nM) for 24 and 48 h, analyzing apoptosis by Annexin-V/PI staining with t test statistic assay. (Mean ± SD, n = 3, *p < 0.05, **p < 0.01, ***p < 0.001 compared with control group; # p < 0. 05, ## p < 0.01 compared with single agent group. (C) Cell lines were subjected to indicate treatments and protein lysates were performed with immunoblotting, incubating with PARP, caspase3, caspase9, cleaved-PARP, cleaved-caspase3, cleaved-caspase9, Bax and Bcl-2 antibodies. (PDF 191 kb) [file 13045_2016_303_MOESM3_ESM.pdf]

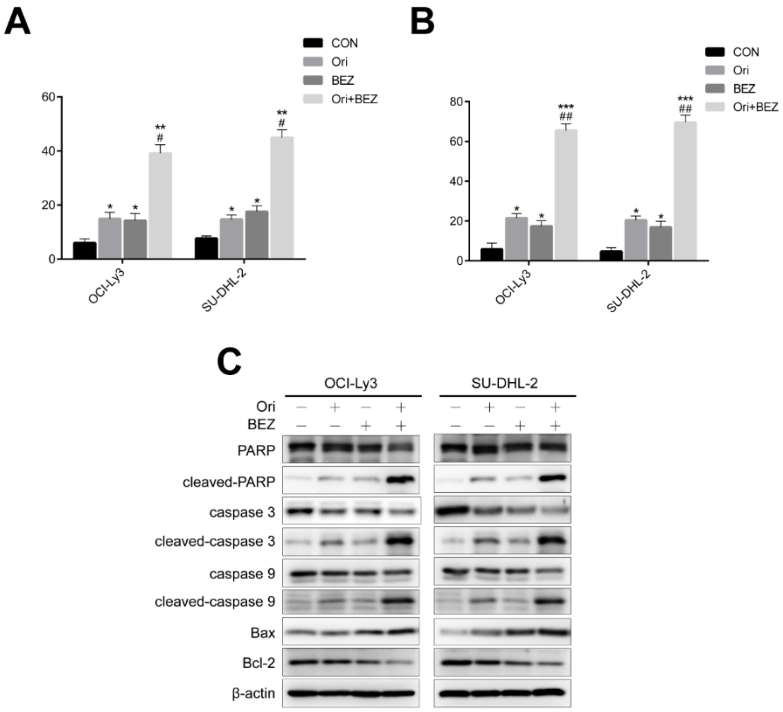

Additional file 3: Oridonin combined with NVP-BEZ235 significantly increased the apoptosis on non-GCB DLBCL cell lines.

(A, B) Cell lines were simultaneously treated with oridonin (2 μM) and NVP-BEZ235 (25 nM) for 24h and 48 h, analyzing apoptosis by Annexin-V/PI staining with t test statistic assay. (Mean ± S.D., n = 3,* $p < 0.05$, ** $p < 0.01$, *** $p < 0.001$ compared with control group; # p < 0. 05, ## $p < 0.01$ compared with single agent group. **(C)** Cell lines were subjected to indicated treatments and protein lysates were performed with immunoblotting, incubating with PARP, caspase3, caspase9, cleaved-PARP, cleaved-caspase3, cleaved-caspase9, Bax and Bcl-2 antibodies.
