# Supplementary material for: Synergistic effect of oridonin and a PI3K/mTOR inhibitor on the non-germinal center B cell-like subtype of diffuse large B cell lymphoma
Source: J Hematol Oncol. 2016 Aug 23;9(1):72. doi: 10.1186/s13045-016-0303-0 (PMC4995739; doi:10.1186/s13045-016-0303-0)
Supplement: Additional file 4: — The effect of oridonin and NVP-BEZ235 on cell cycle. (A) Cells were treated with drugs for 48 h with the dosages indicated in Fig 2. Cell cycle assay was assessed with cytometry and Modfit LT. The percentage of G0 and G1phase cells is respectively shown in the left bottom and left top corner of each panel. (B) The number of G0/G1 cells was determined using quantitative FACS analysis. Each column represents the mean ± SD (n = 3). Statistical analysis was performed using the Student’s t test. NS not significant; *p < 0.05, **p < 0.01 compared with the control group. (PDF 295 kb) [file 13045_2016_303_MOESM4_ESM.pdf]

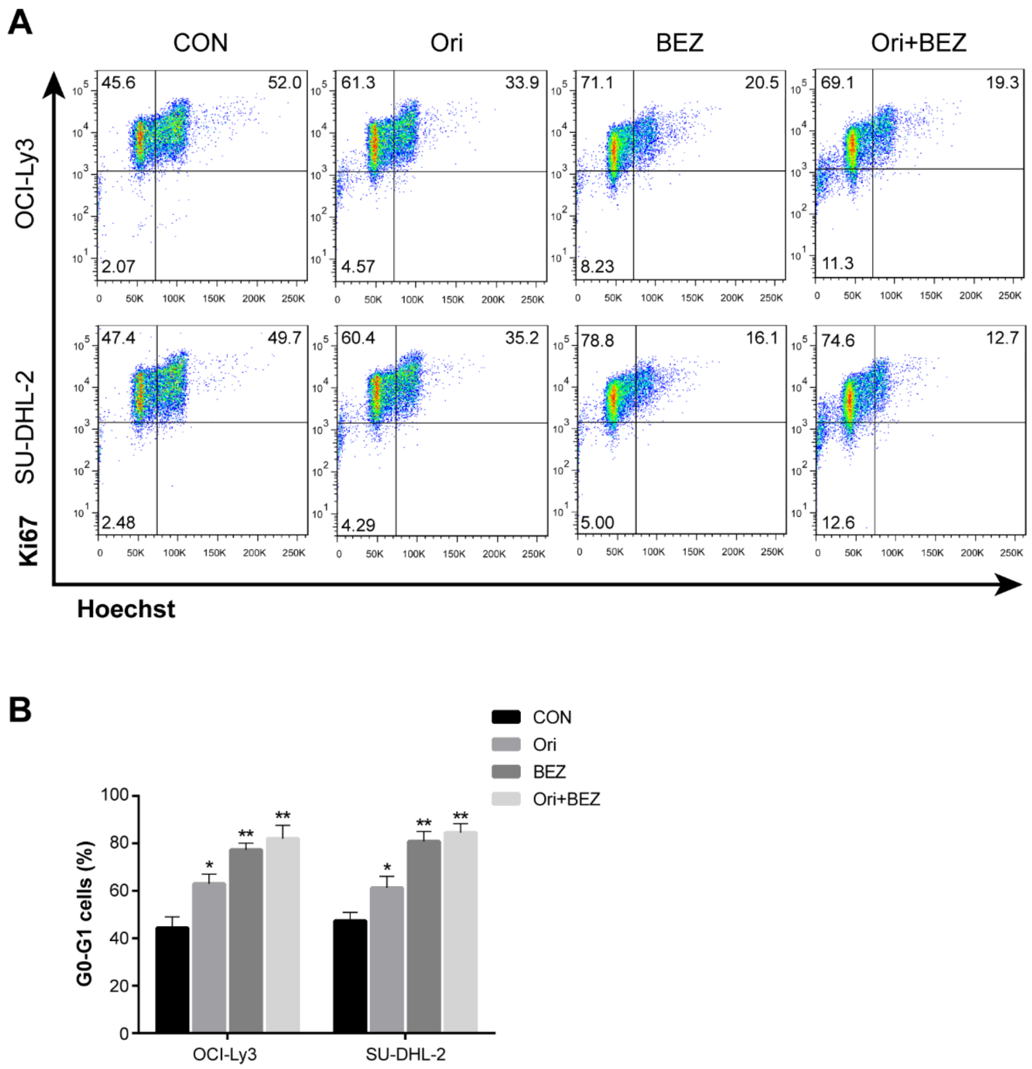

Additional file 4: The effect of oridonin and NVP-BEZ235 on cell cycle.

(A) Cells were treated with drugs for 48 h with the dosages indicated in Fig 2. Cell cycle assay was assessed with cytometry and Modfit LT. The percentage of G0 and G1phase cells is respectively shown in the left bottom and left top corner of each panel.

(B) The number of G0/G1 cells was determined using quantitative FACS analysis. Each column represents the mean ± SD (n = 3). Statistical analysis was performed using the Student's t test. NS, not significant; *P < 0.05, **P < 0.01 compared with the control group.
