# Supplementary material for: Synergistic effect of oridonin and a PI3K/mTOR inhibitor on the non-germinal center B cell-like subtype of diffuse large B cell lymphoma
Source: J Hematol Oncol. 2016 Aug 23;9(1):72. doi: 10.1186/s13045-016-0303-0 (PMC4995739; doi:10.1186/s13045-016-0303-0)
Supplement: Additional file 5: — Co-treatment triggered ROS generation which mediated non-GCB DLBCL cell apoptosis. (A) Cell lines were simultaneously treated with oridonin (2 μM) and NVP-BEZ235 (25 nM) for 48 h, FACS quantitative analysis of DCF-DA was used to detect ROS with t test statistic assay. (Mean ± SD, n = 3, *p < 0.05, **p < 0.01 compared with control group. (B) NAC pretreatment attenuated co-treatment-induced protein expression levels of γH2AX, cleaved-caspase 3 and cleaved-caspase 9. (C) Pretreatment of co-treatment group cells with NAC (5 mM) and Z-DEVD-FMK (10 μM), respectively, for 48 h, analyzing apoptosis by Annexin-V/PI staining. (PDF 404 kb) [file 13045_2016_303_MOESM5_ESM.pdf]

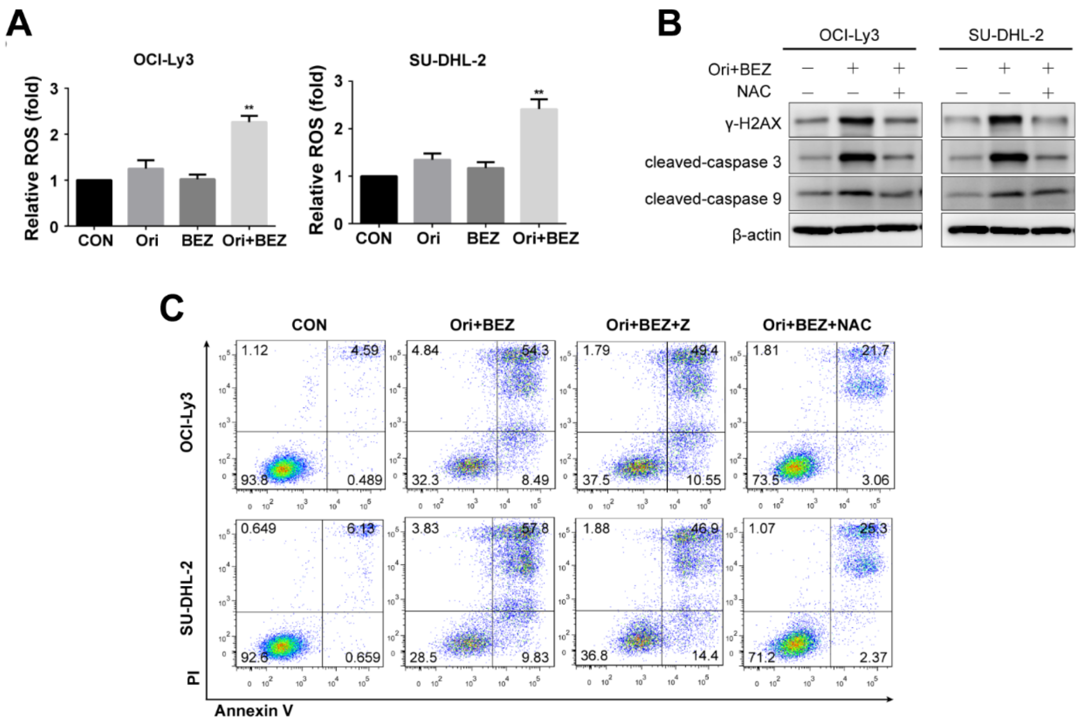

Additional file 5: Cotreatment triggered ROS generation which mediated non-GCB DLBCL cell apoptosis.

(A) Cell lines were simultaneously treated with oridonin (2 μM) and NVP-BEZ235 (25 nM) for 48 h, FACS quantitative analysis of DCF-DA was used to detect ROS with t test statistic assay. (Mean ± S.D., n = 3,* $p < 0.05$, ** $p < 0.01$ compared with control group. **(B)** NAC pretreatment attenuated cotreatment-induced protein expression levels of γH2AX, cleaved-caspase 3 and cleaved-caspase 9. **(C)** Pretreatment of cotreatment group cells with NAC (5 mM) and Z-DEVD-FMK (10 μM) respectively for 48h, analyzing apoptosis by Annexin-V/PI staining.
